# Supplementary material for: Eliciting local knowledge of ecosystem services using participatory mapping and Photovoice: A case study of Tun Mustapha Park, Malaysia
Source: PLoS One. 2021 Jul 9;16(7):e0253740. doi: 10.1371/journal.pone.0253740 (PMC8270451; doi:10.1371/journal.pone.0253740)
Supplement: S1 File — (DOCX) [file pone.0253740.s001.docx]

**MAKLUMAT UNTUK PESERTA PEMETAAN EKOSISTEM**

**Tajuk Kajian**

[GCRF BLUE COMMUNITIES] Memahami sistem sosio-ekologi Taman Tun Mustapha (TMP), Sabah melalui pengetahuan tempatan

**Pengenalan**

Kajian ini adalah untuk mengenal pasti jurang pengetahuan dalam keadaan sosial, ekonomi dan alam sekitar di TMP, dan memahami bagaimana ekosistem marin kini atau bakal digunakan oleh masyarakat tempatan dan sektor perniagaan. Hasil dari kajian ini akan membantu merangka dan melaksanakan pelan pengurusan TMP. Oleh itu, kajian ini akan memetakan ekosistem dan perkhidmatannya di TMP, di samping mengenal pasti ancaman kepada ekosistem tersebut melalui perbincangan kumpulan focus (FGD).

**Objektif**

Tujuan kajian ini adalah untuk memahami situasi sosio-ekologi di TMP melalui pengetahuan masyarakat tempatan dengan:

1. Mengenal pasti jenis-jenis habitat di TMP dan aktiviti di dalamnya yang akan memberi maklumat mengenai perkhidmatan ekosistem
2. Menghasilkan data asas sosio-ekologi di TMP yang akan membantu merangka pelan pengurusan TMP

**Kaedah Kajian dan Kesulitan Rekod**

Kamu akan ditanya soalan mengenai (i) diri kamu, (ii) jenis-jenis habitat di TMP, dan (iii) pengetahuan kamu mengenai perkhidmatan ekosistem di TMP. Jawapan kamu akan digunakan untuk menghasilkan matriks bekalan dan permintaan perhidmatan ekosistem di TMP. Matriks tersebut akan memberi maklumat mengenai (i) potensi pelan pengurusan berdasarkan ekosistem untuk kawasan marin yang dilindungi, dan (ii) untuk membantu penambahbaikan pelan pengurusan TMP terkini.

Sesi ini akan direkod melalui penulisan dan rakaman suara. Sila ambil perhatian:

- Penyertaan kamu dalam kajian ini adalah secara sukarela.
- Kamu boleh menolak permintaan untuk menyertai atau menarik diri dari kajian ini pada bila-bila masa.
- Sekiranya terdapat soalan yang sensitif, kamu boleh memilih untuk tidak menjawab atau menghentikan perbincangan tersebut.
- Maklumat peripadi kamu adalah sulit dan tidak akan dikongsi secara awam melainkan diminta oleh pihak berkuasa.
- Kamu boleh menggunakan nama samaran sekiranya kamu tidak selesa dengan penggunaan nama sebenar kamu.
- Dengan mengisi borang persetujuan ini, kamu bersetuju dengan pengunaan data hasil dari perbincangan ini untuk analisis data dan segala bentuk penerbitan termasuklah report, pelan pengurusan, polisi, artikel kajian dan sebagainya.

**Maklumat lanjut dan butiran perhubungan:**

1. Dr. Goh Hong Ching

Universiti Malaya, Kuala Lumpur

Emel: [gohhc@um.edu.my](mailto:gohhc@um.edu.my)

1. Dr. Amy Then Yee Hui

Universiti Malaya, Kuala Lumpur

Emel: [amy_then@um.edu.my](mailto:amy_then@um.edu.my)

1. Encik Affendi bin Yang Amri

Universiti Malaya, Kuala Lumpur

Emel: [affendi@um.edu.my](mailto:affendi@um.edu.my)

1. Encik Kamal Solhaimi Fadzil

Universiti Malaya, Kuala Lumpur

Emel: [kamal@um.edu.my](mailto:kamal@um.edu.my)

1. Dr. Lim Voon Ching

Universiti Malaya, Kuala Lumpur

Emel: [vclim@um.edu.my](mailto:vclim@um.edu.my)

1. Cik Sofia Johari

Blue Communities Research Programme, Malaysia

Emel: [sofia2611@gmail.com](mailto:sofia2611@gmail.com)

**Aduan**

Sekiranya kamu mempunyai persoalan dan aduan mengenai kajian ini dan kamu tidak ingin berhubung dengan penyelidik yang disenaraikan di atas, kamu juga boleh menghubungi:

| Jawatankuasa | University of Malaya Research Ethics Committee (UMREC) |
| --- | --- |
| Telefon | 03-79677022 (ext : 2369) |
| Emel | umrec@um.edu.my |
| Alamat | **Pusat Perkhidmatan Penyelidikan (PPP)**  Level 2,  Institut Pengurusan & Perkhidmatan Penyelidikan (IPPP)  University of Malaya  50603 Kuala Lumpur, Malaysia |
