# Supplementary material for: Eliciting local knowledge of ecosystem services using participatory mapping and Photovoice: A case study of Tun Mustapha Park, Malaysia
Source: PLoS One. 2021 Jul 9;16(7):e0253740. doi: 10.1371/journal.pone.0253740 (PMC8270451; doi:10.1371/journal.pone.0253740)
Supplement: S2 File — (DOCX) [file pone.0253740.s002.docx]

**Tajuk Kajian**

“Citizen Science Photovoice” (No. Rujukan Etika: UM.TNC2/UMREC – 465)

**Pengenalan**

Projek penyelidikan Blue Communities, Universiti Malaya ini bertujuan untuk memetakan habitat marin di Taman Tun Mustapha (TMP) dan memahami bagaimana masyarakat persisiran pantai menggunakan ekosistem marin tersebut melalui teknik fotografi “Photovoice”. Hasil penyelidikan ini dikongsi dengan Taman-Taman Sabah untuk menyumbang kepada pelan pengurusan TMP.

**Objektif dan Faedah**

Tujuan projek ini adalah untuk memahami hubungan antara masyarakat persisiran pantai dengan habitat marin serta persisiran pantai di TMP. Projek ini akan:

1. Mengenal pasti lokasi habitat marin di TMP (bakau, pantai, rumput laut, batu karang)
2. Mengenal pasti bagaimana masyarakat persisiran pantai menggunakan habitat marin tersebut untuk kehidupan, kesihatan, adat dan sebagainya
3. Melibatkan belia-belia di TMP dalam projek penyelidikan *“Citizen Science”*
4. Memupuk semangat cinta alam sekitar dalam kalangan belia dan mendorong mereka menjaga TMP bersama

Faedah-faedah yang peserta boleh terima dari projek penyelidikan ini adalah:

1. Meluahkan pendapat dan pandangan komuniti kepada pihak berkepentingan
2. Meningkatkan kesedaran isu alam sekitar dan komuniti
3. Meningkatkan kesedaran tentang keindahan tersembunyi di TMP
4. Sumbangan kepada penyelidikan Blue Communities, Universiti Malaya dan usaha mempromosikan TMP oleh Taman-Taman Sabah
5. Mempelajari teknik-teknik fotografi
6. Mendapat hasil dari penjualan poskad, buku gambar dan produk lain

**Kaedah Kajian**

- Peserta akan terlibat dalam satu perbincangan berkumpulan bersama peserta lain.
- Peserta akan menjawab soalan-soalan dalam borang yang diberi oleh Blue Communities.

**Kesulitan Rekod**

- Penyertaan peserta dalam aktiviti ini adalah secara sukarela.
- Peserta boleh menarik diri dari aktiviti ini pada bila-bila masa.
- Gambar yang diambil oleh peserta untuk projek ini dimiliki oleh peserta dan Blue Communities.
- Sekiranya terdapat gambar yang membuatkan peserta kurang selesa, peserta berhak untuk memadam dan tidak berkongsi foto tersebut dengan Blue Communities.
- Maklumat peripadi peserta adalah sulit dan tidak akan dikongsi secara awam melainkan diminta oleh pihak berkuasa.
- Peserta boleh menggunakan nama samaran ketika bengkel berlangsung sekiranya peserta tidak selesa dengan penggunaan nama sebenar.
- Hasil dari bengkel ini akan digunakan untuk segala bentuk penerbitan termasuklah report, pelan pengurusan, polisi, artikel kajian dan sebagainya.
- Pihak penyelidik Blue Communities dan Universiti Malaya tidak bertanggungjawab terhadap sebarang bahaya atau kerugian yang mungkin disebabkan oleh peserta atau berkaitan dengan penyertaan peserta ketika penyelidikan ini.

**Maklumat lanjut dan butiran perhubungan:**

| **1. Nama :** Encik Kamal Solhaimi Fadzil  **Jawatan :** Penyelidik Bersama  **Telefon :** 016-2051656  **Emel :** kamal@um.edu.my  **Institusi :** Universiti Malaya | **2. Nama :** Dr Lim Voon Ching  **Jawatan :**Felo Penyelidik Pasca-Doktoral  **Telefon :** 012-9755977  **Emel :** vclim@um.edu.my  **Institusi :** Universiti Malaya |
| --- | --- |
| **3. Nama** : Dr Goh Hong Ching  **Jawatan** : Ketua Projek Bersama 1  **Emel** : gohhc@um.edu.my  **Institusi** : Universiti Malaya | **4. Nama :** Eva Vivian Justine  **Jawatan :** Pembantu Lapangan  **Telefon :** 019-873 7547  **Emel :** evavivian05@gmail.com  **Institusi :** Universiti Malaya |

**Aduan**

Jika anda mempunyai sebarang pertanyaan berkenaan projek penyelidikan ini, yang anda tidak ingin berbincang dengan penyelidik yang tersenarai di atas, anda boleh menghubungi:

| Jawatankuasa | University of Malaya Research Ethics Committee (UMREC) |
| --- | --- |
| Telefon | 03-79677022 (ext : 2369) |
| Emel | umrec@um.edu.my |
| Alamat Surat-menyurat | **Pusat Perkhidmatan Penyelidikan (PPP)**  Level 2,  Institut Pengurusan & Perkhidmatan Penyelidikan (IPPP)  University of Malaya  50603 Kuala Lumpur, Malaysia |
