# Supplementary material for: Eliciting local knowledge of ecosystem services using participatory mapping and Photovoice: A case study of Tun Mustapha Park, Malaysia
Source: PLoS One. 2021 Jul 9;16(7):e0253740. doi: 10.1371/journal.pone.0253740 (PMC8270451; doi:10.1371/journal.pone.0253740)
Supplement: S3 File — (DOCX) [file pone.0253740.s003.docx]

**BORANG PERSETUJUAN UNTUK PEMETAAN EKOSISTEM**

**Tajuk Kajian**

[GCRF BLUE COMMUNITIES] Memahami sistem sosio-ekologi Taman Tun Mustapha, Sabah melalui pengetahuan tempatan (No. Rujukan: UM.TNC2/UMREC – 465)

Dengan mengisi borang ini, saya mengesahkan bahawa penyelidik telah memberi taklimat mengenai penyelidikan ini dan maklumat peserta kepada saya, dan saya secara sukarela bersetuju untuk mengambil bahagian dalam penyelidikan ini dan akan memberi maklumat kepada penyiasat seperti yang diminta.

1. Umur anda : ___________________
2. Jantina anda :  Lelaki  Perempuan
3. Status perkahwinan :  Sudah berkahwin  Bujang
4. Bilangan ahli keluarga yang anda perlu menjaga: ___________________
5. Adakah anda perlu menjaga keluarga anda : Ya  Tidak
6. Apakah sumber pencarian utama anda? ___________________
7. Apakah tahap pendidikan anda? ___________________
8. Apakah suku kaum anda? ___________________
9. Tarikh hari ini ___________________
10. Sila tanda tangan di sini ___________________

Nama samaran peserta:

Tandatangan penyelidik: Tarikh:
