# Supplementary material for: Eliciting local knowledge of ecosystem services using participatory mapping and Photovoice: A case study of Tun Mustapha Park, Malaysia
Source: PLoS One. 2021 Jul 9;16(7):e0253740. doi: 10.1371/journal.pone.0253740 (PMC8270451; doi:10.1371/journal.pone.0253740)
Supplement: S5 File — (DOCX) [file pone.0253740.s005.docx]

**Tajuk Kajian**

“Citizen Science Photovoice” (No. Rujukan Etika: UM.TNC2/UMREC – 465)

**Untuk dilengkapkan oleh peserta**

Dengan melengkapkan borang ini, saya bersetuju bahawa:

1. Pihak Blue Communities telah memberi taklimat dan maklumat berkenaan penyelidikan yang dijalankan kepada saya
2. Saya telah menerima lampiran maklumat untuk peserta
3. Saya telah membaca dan memahami semua syarat berkaitan dengan penyertaan saya dalam penyelidikan ini
4. Saya bersetuju secara suka rela untuk mengambil bahagian dalam kajian ini dan memberi maklumat yang diperlukan oleh para penyelidik
5. Saya bersetuju dengan pengunaan data hasil dari bengkel ini untuk analisis data dan segala bentuk penerbitan termasuklah report, pelan pengurusan, polisi, artikel kajian dan sebagainya
6. Kecuali bagi kerosakan yang berlaku akibat daripada perlakuan cuai atau niat jahat penyelidik, saya dengan ini melepaskan pihak Blue Communities dan Universiti Malaya daripada segala tanggungan yang dikaitkan, yang timbul atau berkaitan dengan penyertaan saya serta, saya juga bersetuju untuk melepaskan penyelidik dari sebarang bahaya atau kerugian yang mungkin disebabkan oleh saya ketika penyelidikan ini.

Saya dengan ini mengakui telah membaca dan memahami pernyataan-pernyataan di atas dan menandatangani borang ini secara suka rela.

Nama : ___________________________________

Tarikh : ___________________________________

Tandatangan : ___________________________________

**Untuk dilengkapkan oleh ibubapa/penjaga bagi peserta yang berumur 18 tahun ke bawah**

Nama ibubapa/penjaga : ____________________________

Hubungan dengan peserta : ____________________________

Tarikh : ____________________________

Tandatangan ibubapa/penjaga : ____________________________

**Untuk dilengkapkan oleh penyelidik Blue Communities**

Nama penyelidik : ____________________________

Tarikh : ____________________________

Tandatangan penyelidik : ____________________________

| **TARIKH:** | **PERSATUAN:** |
| --- | --- |
| **NAMA:** | **UMUR:** |
| **JANTINA:** | **ETNIK:** |
| **PEKERJAAN:** | |

1. ID gambar (penyelidik tolong tulis):__________________________________________
2. Tandakan [X] soalan penyelidikan yang gambar anda jawab

| 1. Apakah yang penting bagi anda, keluarga dan komuniti anda di laut, rumput laut, batu karang, bakau dan pantai? |  |
| --- | --- |
| 1. Apakah perubahan di laut, rumput laut, batu karang, bakau dan pantai yang telah memberi kesan kepada anda, keluarga dan komuniti anda? |  |
| 1. Apakah harapan anda, keluarga dan komuniti anda untuk masa depan laut, rumput laut, batu karang, bakau dan pantai? |  |
| 1. Apakah yang anda, keluarga dan komuniti anda risaukan tentang masa depan laut, rumput laut, batu karang, bakau dan pantai? |  |

1. Isikan maklumat gambar anda

| Tarikh: |  |
| --- | --- |
| Masa: |  |
| Lokasi: |  |
| Koordinat GPS: |  |

1. Apakah naratif / cerita di sebalik gambar anda?

|  |
| --- |
|  |
|  |
|  |
|  |

1. Apakah *ecosystem services* yang terdapat / terjadi dalam gambar anda?

|  |
| --- |
|  |
|  |
|  |
|  |

1. Kenapa anda mengambil gambar ini?

|  |
| --- |
|  |
|  |
|  |
|  |

1. Apakah ancaman terhadap hidup anda dan komuniti anda dalam gambar ini?

|  |
| --- |
|  |
|  |
|  |
|  |

1. Bagaimana gambar ini boleh menyumbang kepada masa depan yang lebih baik?

|  |
| --- |
|  |
|  |
|  |
|  |

1. Bagaimana habitat marin dan laut (contoh: batu karang, rumput laut, bakau dan pantai) dalam gambar ini memberi manfaat kepada kehidupan harian anda?

|  |
| --- |
|  |
|  |
|  |
|  |

1. Pada pendapat anda, bagaimana habitat marin dan laut dalam gambar ini boleh dijaga lagi?

|  |
| --- |
|  |
|  |
|  |
|  |

1. Adakah anda mempunyai pandangan atau komen yang anda ingin berkongsi dengan kami?

|  |
| --- |
|  |
|  |
|  |
|  |
