# Supplementary material for: Eliciting local knowledge of ecosystem services using participatory mapping and Photovoice: A case study of Tun Mustapha Park, Malaysia
Source: PLoS One. 2021 Jul 9;16(7):e0253740. doi: 10.1371/journal.pone.0253740 (PMC8270451; doi:10.1371/journal.pone.0253740)
Supplement: S1 Table — (DOCX) [file pone.0253740.s006.docx]

**Supplementary Tables**

Table S 1: Questions in the three parts of the EM’s FGD which were asked in a sequential manner. The purpose of the questions was to seek information on marine ecosystem in TMP that were perceived to be important for providing certain provisioning, cultural and regulating services to local communities. Participants responded to the questions by pasting provided stickers (of habitats and human activities) and drawing additional features on a map of TMP. Certain questions required participants to respond verbally..

| **Part** | **Questions** | **Significance** | **Mode of response** |
| --- | --- | --- | --- |
| 1. | (Participants were shown photographs of beaches [i.e. sandy, muddy and rocky], corals, mangroves and seagrasses)  *Have you seen these habitats in TMP or near your village in TMP?* | Identify habitats associated with marine ecosystem in TMP and allow participants to familiarise with these habitats | Paste stickers of habitats and draw on map |
| 2. | *Do you earn income and obtain food from animals and plants in the habitats that you mentioned?*  *If yes, can you explain how and where you obtained them? Can you tell me their names, how you use them and where you usually fish?* | Identify provisioning services in TMP | Paste stickers of activities and draw on map  Verbal response |
|  | *Is this the only way to get the food or income?* | Validate information from previous question and seek for new information | Verbal response |
|  | *Beside food and source of income, do you use the habitats that you mentioned for other specific use?*  *If yes, can you explain in detail and where is the location?* | Identify other provisioning services (e.g. material for tool or clothes) in TMP | Paste stickers of activities and draw on map  Verbal response |
|  | *Do you always obtain your food and other material this way? Or will you only do this when you have no options left?* | Validate information from previous question and seek for new information | Verbal response |
|  | *Do you do any recreational activities in the habitats that you mentioned by yourself or with your family?* | Identify cultural services (i.e. mental well-being) in TMP | Paste stickers of activities and draw on map  Verbal response |
|  | *Do you learn any skills or tradition from your elders in the habitats that you mentioned?*  *If yes, can you explain the activities, how you benefit from and where did you do those activities?* | Identify cultural services (i.e. knowledge and tradition) in TMP | Paste stickers of activities and draw on map  Verbal response |
|  | *Are the habitats that you mentioned important to your customary belief or community?*  *If yes, can you explain in detail and where is the site?* | Identify cultural services (i.e. religious and customary belief) in TMP | Paste stickers of activities and draw on map  Verbal response |
|  | *How do you feel when you are in the habitats that you mentioned?*  *How will you feel if the habitats are destroyed or lost?* | Identify cultural services (i.e. spiritual and mental well-being) in TMP | Verbal response |
|  | *Are there any habitats that have been destroyed? Can you show us where? Do you feel it’s different now after the habitats were destroyed or lost?* | Identify regulating services (i.e. colder or warmer temperature, rise in erosion) and potential anthropogenic threats in TMP | Paste stickers of activities and draw on map  Verbal response |
|  | *Do you have anything else to add?* | Validate information from previous question and seek for new information | Paste stickers of activities and draw on map  Verbal response |
| 3. | *You mentioned [a specific habitat] just now. Is it important and why do you think so?* | Validate information from previous question and seek for new information | Verbal response |
