# Supplementary material for: Eliciting local knowledge of ecosystem services using participatory mapping and Photovoice: A case study of Tun Mustapha Park, Malaysia
Source: PLoS One. 2021 Jul 9;16(7):e0253740. doi: 10.1371/journal.pone.0253740 (PMC8270451; doi:10.1371/journal.pone.0253740)
Supplement: S2 Table — (DOCX) [file pone.0253740.s007.docx]

**Supplementary Tables**

Table S 2: Questions for Photovoice activity where participants need to answer based on their chosen photograph and caption.

| **Questions** | **Remarks** |
| --- | --- |
| What is the story behind your **P**hoto? | mnemonic PHOTO (Hergenrather, Rhodes, Cowan, Bardhoshi, & Pula, 2009) |
| What are the ecosystem services **H**appening in your photo? |  |
| Why did you take a photo **O**f this? |  |
| What are the **T**hreats to your life or your community in this photo? |  |
| How can this picture provide **O**pportunities for things to be better in future? |  |
| How marine habitats could benefit your daily life? | Ecosystem services |
| Based on your opinion, how can the marine habitats be preserved and conserved? | Suggestions for improvement |
| Do you have any thoughts or comments that you would like to share with us? | To capture information that could not be captured with existing method and questions |
